# Ungewöhnliche(r) Differenzialdiagnose oder Bruchsackinhalt einer suspekten rechtsseitigen Leistenhernie

**DOI:** 10.1007/s00104-025-02427-4

**Published:** 2025-12-16

**Authors:** Mohammad Othman Alhomsi, Benjamin Ullrich, Christine Rose, Johann Jakob Wendler, Christoph Paasch, Roland Croner, Frank Meyer

**Affiliations:** 1 Klinik für Allgemein‑, Viszeral‑, Gefäß- und Transplantationschirurgie, Otto-von-Guericke-Universität mit Universitätsklinikum, Leipziger Str. 44, 39120 Magdeburg, Deutschland; 2 Klinik für Radiologie und Nuklearmedizin, Otto-von-Guericke-Universität mit Universitätsklinikum, Magdeburg, Deutschland; 3 Urologische Praxis am Hasselbachplatz, Magdeburg, Deutschland; 4 Klinik für Allgemein- und Viszeralchirurgie, Universitätsklinikum Brandenburg, Brandenburg, Deutschland

## Falldarstellung

### Anamnese

Ein 62-jähriger Mann stellte sich über die Notaufnahme mit einer stationären Einweisung bei Druckschmerz und Fremdkörpergefühl in der rechten Leiste vor. Eigenanamnestisch war beim Patienten ein gastrointestinaler Stromatumor (GIST) des Magens vor 6 Jahren zu eruieren, der mittels einer partiellen Magenresektion versorgt worden war. Die aktuelle Medikation umfasste Imatinib im adjuvanten Setting seit GIST-Manifestation und -Versorgung. Miktionsbeschwerden gab der Patient nicht an.

### Klinischer Befund

Es wurde eine halbfaustgroße Schwellung im Sinne einer nicht eingeklemmten Leistenhernie rechts-inguinal bei mäßig dolenter Palpation am äußeren Leistenring im Stehen und beim Husten (mit positivem Anprallphänomen) gefunden.

### Diagnostik

Laborchemisch zeigten sich normwertige Entzündungsparameter (Leukozytenzahl, CrP) sowie eine regelrechte Gerinnung (Quick, Thrombozytenzahl) und Elektrolyte im Normbereich.

In der Abdomensonographie kam eine rechtsseitige Leistenhernie zur Darstellung. Zudem zeigte sich eine luminäre Struktur, nicht als Darminhalt imponierend. Es schloss sich eine Computertomographie des Abdomens an.

## Wie lautet Ihre Diagnose?

### … Fortsetzung der „Diagnostik“

**Abb. 1:**
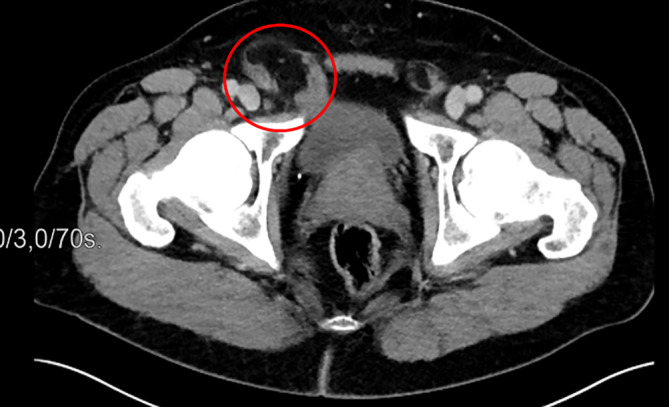
CT – Transversalscan (*roter Kreis*: umfährt die Ausdehnung der Inguinalhernie rechts mit Harnblasendivertikel im Bruchsack)

In der Computertomographie (CT) des Abdomens kam ein Harnblasendivertikel im Bruchsack zur Darstellung (Abb. 1 und 2).

**Abb. 2:**
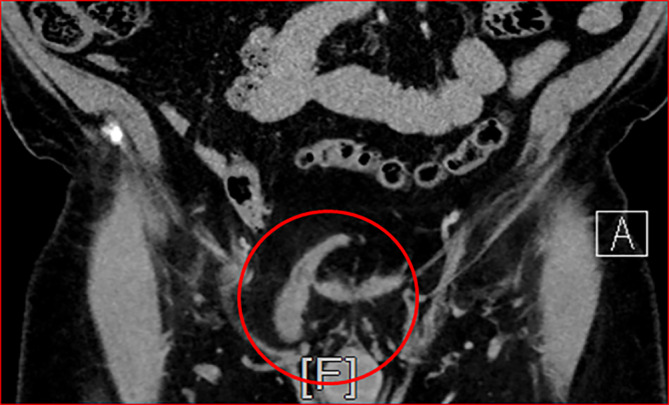
CT – Koronarscan (*roter Kreis*: umfährt die Ausdehnung der Inguinalhernie rechts mit Harnblasendivertikel im Bruchsack)

### Therapie

Es wurde eine Leistenhernienversorgung in Lichtenstein-Technik vorgenommen. Hierbei wurde ein ProGrip^®^-Netz (Fa. Medtronic GmbH, Deggendorf, Deutschland), eingesetzt. Intraoperativ zeigte sich nach Bruchsackeröffnung ein Harnblasendivertikel als derbe, muskulöse Wandstruktur imponierend.

### Verlauf

Der intra- und postoperative Verlauf waren frei von eingriffsspezifischen Komplikationen. Der Patient konnte am vierten postoperativen Tag entlassen werden.

### Differenzialdiagnose

Leistenhernie mit intraabdominellem Fettgewebe, Darminhalt (z. B. Littré-Hernie, Amyand-Hernie, de-Garangeot-Hernie), Mesosalpinx oder Peritonealkarzinose im Bruchsack; Hernia fermoralis, Lipom (nicht zutreffend bei Schnittbildgebung), Atherom, vergrößerte inguinale Lymphknoten.

### Definitive Diagnose

Rechtseitige Leistenhernie mit Harnblasendivertikel im inguinalen Bruchsack

## Diskussion

### Krankheitsbeschreibung

Der mögliche Bruchsackinhalt begrenzt sich nicht nur auf Peritoneum, Fettgewebe, Darm, sondern in seltenen Fällen eben auch Harnblasendivertikel, manifeste Tumorläsionen einer Peritonealkarzinose [1], Appendix vermiformis (Amyand-Hernie [5]), Meckel-Divertikel [3, 8] oder Eileiter/Eierstock.

Mit dem präsentierten Patienten wird ein eigener Fall mit Harnblasenanteilen im Bruchsack einer indirekten Hernie beschrieben [4], wie auch von Gurer et al. in *Hernia* 2006 berichtet [6].

Aufgrund der meist unspezifischen Symptomatik und der niedrigen Prävalenz (kongenitale Harnblasendivertikel kommen in 1,7 % der Fälle und echte Blasendivertikel mit ca. 0,7 % vor) kann diese Pathologie nicht selten präoperativ unerkannt bleiben. Die korrekte Diagnosestellung sowie die adäquate chirurgische Versorgung erfordern daher ein hohes Maß an Aufmerksamkeit seitens des behandelnden Teams. Patienten berichten über unspezifische Beschwerden im Leistenbereich, Dysurie, Pollakisurie, Harnverhalt oder postmiktionales Nachtröpfeln. Gelegentlich kommt es zu rezidivierenden Harnwegsinfektionen. Pathognomonisch, jedoch selten erkannt, ist das sog. *zweizeitige Wasserlassen*, bei dem Patienten nach initialer Blasenentleerung durch Kompression der Hernie weiteren Urin entleeren. Beim berichteten Patienten waren dahingehend keine anamnestischen Angaben zu erheben.

**Diagnose:** Rechtseitige Leistenhernie mit ungewöhnlichem/r Bruchsackinhalt und oder Differenzialdiagnose

Die präoperative Bildgebung spielt eine entscheidende Rolle für die Diagnosestellung. Während die sonographische Darstellung erste Hinweise (mit kommunizierendem Lumen) liefern kann, ist präoperativ ein CT-Abdomen zur weiterführenden Diagnostik wie im vorliegenden Fall sinnvoll zur klärenden (bzw. bestätigenden) Erkennung des Bruchsackinhaltes und Vermeidung der intraoperativen und postoperativen Komplikationen [10]. Konkret wäre zusätzlich im berichteten Kasus die computertomographische Urographie (CT-Urographie) als Mittel der Wahl zur Erstellung eines Zystodivertikulogramms überlegenswert gewesen. Sie erlaubt eine exakte Darstellung der Hernie sowie des Blasendivertikels und erleichtert somit die Operationsplanung.

Die ausgelassene bzw. fehlende präoperative Identifikation eines blasenhaltigen Leistenbruchs birgt das Risiko einer intraoperativen Blasenverletzung mit potenziell schwerwiegenden postoperativen Komplikationen.

### Therapie

Das operative Management dieser speziellen Hernienform erfordert eine interdisziplinäre Zusammenarbeit zwischen Chirurgie und Urologie. Das primäre Therapieziel besteht in der vollständigen Reposition des Blasendivertikels sowie in der adäquaten Rekonstruktion der Leistenregion.

Je nach Konstitution des Divertikels und Zustand der Blasenwand sind unterschiedliche Betrachtungen anzustellen: So sind Pseudodivertikel gegenüber echten Divertikeln dünnwandiger und schwieriger von der Umgebung abzugrenzen.

### Spezifische fallbezogene Aspekte

Die im klinischen Alltag eher seltene Leistenhernie mit vorwölbendem Harnblasendivertikel stellte im präsentierten Fallbeispiel eine überraschende Diagnose dar, die eindeutig mittels Sonographie, CT und chirurgischem Eingriff hinsichtlich der (Differenzial‑)Diagnose geklärt werden konnte.

Klinisch lagen Schwellung und Schmerzen in der rechten Leiste vor. Dabei konnte die Schwellung nicht suffizient reponiert werden.

Sonographie und komplementäre CT (insbesondere für unklare Befundaspekte bzw. für Befunderhärtung bzw. für definitiven -ausschluss) stellten diagnostisch mit ihrer Spezifität die geeigneten „Tools“ dar, um die Diagnose zu stellen sowie eine mögliche Inkarzeration auszuschließen, wenn die Befundkonstellation hierauf auch nicht klinisch massiv suspekt war.

Intraoperativ wurde die Alteration des Bruchsackinhaltes sensibel vermieden – die eher derb und muskulär erscheinende Wand der luminalen „Struktur“ im Bruchsack sprach eher nicht für das Intestinum (wobei aus systematisierender Sicht eher ein echtes [kongenitales] Blasendivertikel anzunehmen war).

Bei symptomatischen Blasendivertikeln kann – aus fachspezifisch urologischer Sicht – eine operative Divertikelresektion angezeigt sein. Der Eingriff gilt jedoch als anspruchsvoll, da dabei sowohl die nervale und vaskuläre Versorgung des betroffenen Blasenquadranten als auch eine mögliche direkte oder indirekte Involvierung des Harnleiters und dessen Ostiums berücksichtigt werden müssen. Dies erfordert eine sorgfältige präoperative Diagnostik und ggf. eine intraoperative Ureterdarstellung.

Der hier dargestellte Fallbericht beschreibt eine Herniation von Blasenwandanteilen im Rahmen eines hernierten, echten Blasendivertikels. Die präoperative Identifizierung des Divertikels sowie die Klärung der anatomischen Beziehung zum Leistenkanal sind entscheidend für die operative Planung. In Extremfällen können jedoch noch komplexere anatomische Strukturen in eine Leistenhernie verlagert sein. So wurde beispielsweise in der Literatur auch über die Herniation einer Niere in den Leistenkanal berichtet [13].

### Differenzialdiagnose

- Die Kombination aus Leistenhernie und Blasendivertikel wird in der Literatur nur selten beschrieben und stellt für den behandelnden Chirurgen eine diagnostische und operative Herausforderung dar. Für den Fall einer fehlenden präoperativen Diagnosestellung kann es intraoperative Komplikationen oder postoperative Funktionsstörungen zur Folge haben.
- Ebenfalls selten findet man die sog. Littré-Hernie mit einem Meckel-Divertikel (Residuum durch Rückbildungshemmung des Dotterganges, einer Verbindung zwischen Darm und Dottersack) als außergewöhnlichen Bruchsackinhalt [3].
- Im klinischen Alltag weiterhin erwähnenswert ist die Amyand-Hernie [7], gekennzeichnet durch die Appendix vermiformis im Bruchsack, die nach Losanoff und Basson in 4 Subtypen unterteilt ist (physiologische Appendix vermiformis, physiologische Appendix vermiformis innerhalb der Leistenhernie, Hernie mit Appendizitis, Hernie mit perforierter Appendizitis und Hernie mit Komplikationen einschließlich Abszess oder Malignität – zitiert in [12]). Eine weitere Sonderform stellt die de-Garangeot-Appendizitis dar, gekennzeichnet durch eine entzündete Appendix vermiformis im Bruchsack einer Hernia femoralis.
- Die Sonographie als Goldstandard und leitliniengerechte Diagnostik sollte auch in der Lage sein, Mesosalpinx und Tuba uterina als Bruchsackinhalt zu detektieren [11].
- Eine betonte subkutane Venenausprägung an Oberschenkelinnenseite und großen Labien – teils thrombosiert – kann eine irreponible Leisten- bzw. Schenkelhernie imitieren [11].
- Eine irreponible Leistenhernie zeigte sich ebenso bei einem außergewöhnlichen Fall mit Nebennierenrindenkarzinom (NNR-Ca), wo sich ein Peritonealkarzinoseknoten als Bruchsackinhalt darstellte [1]. Neben diversen Bruchsackinhalten einer Hernia inguinalis besteht eine durchaus beachtliche Breite des Spektrums von „Tumor“-assoziierten Differenzialdiagnosen wie Lymphknotenschwellung (benigne/maligne)/Lymphadenopathie, Lipom (eher nicht bildgebend), Samenstrangzyste (Manuskript in Vorbereitung), Weichteilsarkom, Atherom, Abszess, Tuberkulose (Tbc) und Leistenhoden (eher bei Kindern oder aber Patienten aus Teilen der Welt mit geringerem sozialökonomischen Einkommen) – nicht zuletzt kann eine inguinale (flüssigkeitsbedingte) Schwellung von einer manifesten Pankreatitis (i. S. einer imitierten hernienbedingten inguinalen Schwellung) herrühren durch „Abfluss“ von Pankreasflüssigkeit aus der peripankreatischen Region entlang der parakolischen Rinne rechts und links auf der Gerota-Faszie mit Anschluss an den Anulus inguinalis profundus, wie berichtet [2].
  Erwähnenswert ist nicht zuletzt die *supravesikale Hernie* mit dem Ausgangspunkt der Fossa supravesicalis, die beidseits des Ligamentum umbilicale medianum (obliterierter Urachus) liegt und von der Harnblase und Ligamentum umbilicale mediale (obliterierte Umbilikalgefäße) begrenzt wird. Die supravesikale Hernie stellt bei der Präparation eine Herausforderung für den Operateur dar. Sie kann offen oder minimalinvasiv operiert werden [5, 9].

## Fazit für die Praxis

- Der geschilderte Fall verdeutlicht die Bedeutung einer sorgfältigen präoperativen Diagnostik bei Leistenhernien, insbesondere bei atypischen Symptomen oder unklaren Befunden in der Bildgebung in Korrelation mit der klinischen Verdachtsdiagnose.
- Fallserien oder multizentrische Registerstudien könnten helfen, die Prävalenz, klinische Verläufe und optimale Therapieoptionen solcher Sonderformen besser zu verstehen und Leitlinienempfehlungen weiter zu differenzieren.
